# Oocyte Cryopreservation in Oncological Patients: Eighteen Years Experience of a Tertiary Care Referral Center

**DOI:** 10.3389/fendo.2019.00600

**Published:** 2019-09-03

**Authors:** Cristina Specchia, Annamaria Baggiani, Valentina Immediata, Camilla Ronchetti, Amalia Cesana, Antonella Smeraldi, Giulia Scaravelli, Paolo Emanuele Levi-Setti

**Affiliations:** ^1^Division of Gynaecology and Reproductive Medicine, Department of Gynaecology, Humanitas Fertility Center, Humanitas Clinical and Research Institute, Milan, Italy; ^2^ART Italian National Register, National Centre for Epidemiology, Surveillance and Health Promotion, National Health Institute, Rome, Italy; ^3^Department of Obstetrics, Gynaecology and Reproductive Sciences, Yale University, School of Medicine, New Haven, CT, United States

**Keywords:** fertility preservation, cancer, oocyte, vitrification, pregnancy outcome

## Abstract

**Objective:** The aim of the present study is to report our experience on elective women fertility preservation before cancer treatment.

**Study Design:** This is a single-center retrospective observational study, including all patients who underwent elective fertility preservation before oncological treatment between January 2001 and March 2019 at our Institute.

**Results:** Of a total of 568 women who received fertility counseling, 244 (42.9%) underwent 252 oocyte retrieval cycles after controlled ovarian stimulation for cryopreservation. The majority of patients were diagnosed with breast cancer (59.9%), followed by women affected by Hodgkin's and non-Hodgkin's lymphoma (27.4%). A minority comprised patients diagnosed with other malignancies that affected soft tissues (2.8%), ovary borderline type (2.4%), digestive system (1.6%), leukemia (1.6%), uterine cervix (1.2%). The remaining 3.1% were affected by other cancer types. The mean age of the cohort was 31.3 ± 6.4 years and the mean oocyte retrieval was 13.5± 8.4. Of 11 women who returned to attempt a pregnancy, three performed two thawed cycles. We obtained four pregnancies from 24 embryo transfers (Pregnancy Rate 36.4% for couple): two miscarriages and two live births. Overall, 95.7% of oocytes are still in storage.

**Conclusions:** A close collaboration between Cancer and Fertility Center in a tertiary care hospital is essential to provide a good health service in oncological patients. Offering fertility preservation is no longer considered optional and must be included in every therapeutic program for women who receive an oncological diagnosis in their reproductive age. Oocyte cryopreservation appears to be a good opportunity for fertility preservation. Our results, although they are obtained in a small sample, are encouraging, even if only 4.5% of patients returned to use their gametes.

## Introduction

Worldwide the incidence of cancer in women between the age of 15 and 39 is 48.7/100,000, and in women aged between 40 and 44 it is 180.1/100,000 (1), representing 13% of all newly diagnosed neoplasias. Breast, cervical, uterine, thyroid, and ovarian cancers are the most prevalent (2). Thanks to wider screening programs, prompter diagnoses, and more effective therapeutic strategies, the survival rate has nowadays increased (3) and this has led clinicians to focus on long-term quality of life issues including access to motherhood. Firstly, antiblastic therapy may indeed cause infertility as a side effect, due to premature ovarian failure, reducing patient's chances to conceive (4, 5). However, in cancer patients the presence of malignancy and the type of cancer have been suggested as factors that can also affect ovarian function (6). For a better counseling at the time of cancer diagnosis, clinicians should take into account women's desire to have children and a multi-disciplinary team support, including an oncologist, a gynecologist, and a psychological support professional, should be offered (7). Strategies to preserve fertility in women undergoing gonadotoxic treatment include embryo cryopreservation (EC), oocyte cryopreservation (OC) and others still considered experimental in many countries and according to several professional organizations as ovarian tissue cryopreservation (OTC) (8), ovarian transposition and ovarian suppression by gonadotropin-releasing hormone (GnRH) agonist (9–11). Timing of cancer treatment, the specific regimen, cancer typology, presence or absence of a partner, patient's age, body mass index and ovarian reserve status are the main factors for clinicians to record in order to establish the most suitable fertility preservation method (12). A reproductive plan need to implemented and one option not exclude another one: Gn-RH analog can be considered in breast cancer patients, but without excluding oocyte and/or ovarian tissue preservation (13, 14). The Italian law regulating assisted reproduction does not permit the cryopreservation of embryos for infertile couples (15) and although this banning was abolished by the Constitutional Court in 2009 (16), its use in fertility preservation is still unclear in our country. In Humanitas Fertility Center, among the above listed options available for cancer patients, vitrification of MII oocytes is the preferred one, at least for post-pubertal patients. Ovarian tissue cryopreservation is still considered experimental, as it requires a surgical procedure, it needs reimplantation and data on obtained pregnancies are not conclusive, though very promising (17). We propose OTC laparoscopic approach only in cases in which the oncologists don't allow the 10–14 days needed to ovarian stimulation and oocyte retrieval or in cases of pre-pubertal or very young women. Ovarian tissue is the only cryopreservation procedure in which we still use low-freezing. Thanks to improved vitrification techniques, which undoubtedly represent one of the greatest advances made in assisted reproduction in recent years. Oocyte survival rate is sub-optimal with no significant differences in implantation or pregnancy rates between embryos obtained from cryopreserved mature oocytes and fresh oocytes only in selected populations OC requires ~2 weeks, since a controlled ovarian hyperstimulation (COH) is needed prior to the procedure. It is not an option for patients with aggressive cancers that must be treated immediately nor in pre-pubertal children. Scarce evidences are reported in literature for those cancer patients who have returned to use their cryopreserved oocytes once they have overcome all the phases related to their disease (18). The aim of the present study is to report our experience on elective fertility preservation (FP) before cancer treatment in order to increase clinicians' knowledge of the current state of oocyte vitrification as a mean to safeguard oncological patients' fertility and to improve their awareness toward such matter.

## Materials and Methods

### Population

We conducted a retrospective cohort study evaluating data of all fertility preservation treatment cycles in women with cancer at the Fertility Center of Humanitas Research Hospital, Rozzano (Milano), Italy, from January 2001 to March 2019. Ovarian tissue cryopreservation cycles were excluded from the current analysis. We did not have full data on women's baseline ovarian reserve, since the treatment was usually started in a random phase of the given menstruation cycle in which they were referred. The study was conducted in accordance with the internal guidelines of the Humanitas Research Hospital Ethics Committee. Patients who underwent cycles had consented in writing that their medical records could be used for research purposes, as long as anonymity and confidentiality of the medical record was protected.

Referrals for FP were received from the Humanitas Oncology Center as well as other oncologysts and evaluated by the senior medical staff of Humanitas Fertility Center dedicated to oncological fertility preservation. Patients obtained an appointment within a few days. Personnel resources have been allocated to enable scheduling and immediate access to consultations, including on-call clinicians and embryologists during holiday periods. The oncologist provided information requested on the disease stage and planned date of initiation of treatment. As a rule, FP should not cause any delay in starting a planned cancer treatment. Every year a phone call was scheduled to renew and confirm the desire of continuing oocytes storage.

At the time of counseling, the ovarian reserve was evaluated by counting antral follicles by transvaginal ultrasonography performed at any moment of the menstrual cycle and, if possible, by measuring serum concentrations of FSH and, in the past 5 years, also Anti-Mullerian Hormone (AMH) levels.

Most patients underwent a gonadotropin releasing hormone (GnRH) antagonist cycle with recombinant FSH (rFSH) or human menopausal gonadotrophin (hMG) starting possibly in the early follicular phase of the cycle (Day 2–3) or randomly. Ganirelix (Orgalutran, MSD Organon, Oss, Netherlands) or 0.25 mg Cetrorelix (Cetrotide, Merck-Serono, Geneva, Switzerland) was added when a leading follicle reached ≥12 mm. We also prescribed aromatase inhibitor 5 mg daily (Femara, Novartis, NJ, USA) to patients with hormone-dependent breast cancer during the stimulation period starting from the second day (19) of the induction to 7 days after oocyte retrieval. Final oocyte maturation was triggered by subcutaneous injection of 0.25 mg recombinant hCG (Ovitrelle, EMD Serono, MA, USA) or 0.2 mg Triptorelin (Decapeptyl, Ipsen, France) according to local protocols (20) when at least two follicles reached 16 mm in diameter. The use of analogs as triggers has decreased the risk of OHSS (Ovarian Hyperstimulation Syndrome), which can delay the start of chemotherapy after fertility preservation. For the same reason, other stimulation protocols (agonist long protocol, flare protocol) have been abandoned in the last years.

Ultrasound-guided oocyte retrieval was performed 36 h later under deep sedation (21). After collection all the oocytes (germinale vesicle–GV, Metafase I–MI, and Metafase II–MII) were selected and cryopreserved using slow freezing until 2009 (22) and open vitrification technique as described by Kuwayama et al. (23) after 2010. All the vitrification and warming solutions were obtained from Kitazato^®^ (Kitazato, Shizuoka, Japan).

In infertile couples and women that perform elective oocyte freezing for postponing motherhood we usually store only mature (MII) oocytes, but in oncological patients we decided to store even immature oocytes in order to have a chance of future use, even if actually still experimental (24).

All costs of the procedures were covered by the NHS, including the Gonadotropins from August 2016.

### Endometrial Preparation for Embryo Transfer

Shortly after menses the subjects received oral estradiol valerate (EV) (Progynova^®^, 6 mg/day Schering, Madrid, Spain). Approximately 10 days after initiating EV endometrial thickness was measured. Administration of micronized progesterone (P) (600 mg/day), vaginally (Progeffik, Effik Laboratories, France), was initiated 3 days prior to embryo transfer (ET) for Day 3 ETs. For the ET of blastocysts, P was initiated 5 days prior to transfer. If pregnancy was achieved, administration of EV and P was maintained until gestation week 12.

## Results

**Figure 1:**
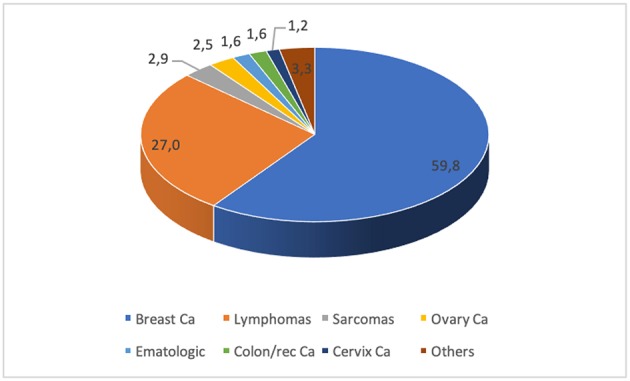
Distribution of malignancies in our population.

**Figure 2:**
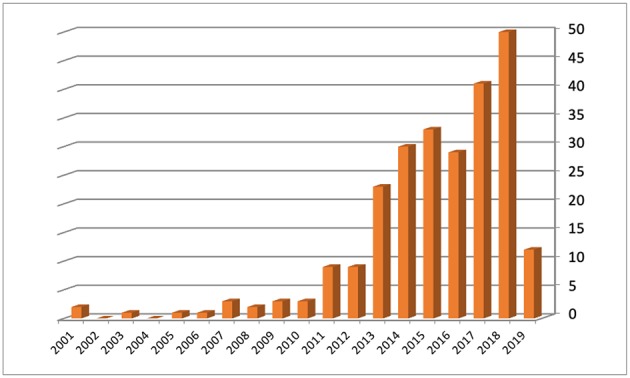
Number of ovulation induction cycles for oocytes cryopreservation per year.

Of 568 women who received fertility counseling, 244 (42.9%) underwent 252 oocyte retrieval cycles after controlled ovarian stimulation for cryopreservation. Most patients were diagnosed with breast cancer (59.8%), followed by women affected by Hodgkin's and non-Hodgkin's lymphoma (27.3%). A minority comprised patients diagnosed with other malignancies (Figure 1). The number of ovulation induction cycles for oocytes cryopreservation followed an increasing trend over the years, due to an emerging attention by oncologists to the patients' desire of childbearing after therapy and a stronger collaboration between our Cancer Center and Fertility Center (Figure 2). The mean age of the cohort was 31.3 ± 6.4 years (range 16–45 years). Among the two main groups, i.e., patients affected by breast cancer and oncoematological diseases, the average age was 34.4 and 25.9, respectively. No serious side effects or complications were observed. The mean number of oocytes retrieved was 13.5 ± 8.4 with a range of 0–40 per patient. The cancellation rate for failure response was 5.1%. The mean number of mature (MII) oocytes stored was 9.5 ± 6.1 with a range of 0–28 per patient (Table 1). Eleven patients (4.5%) returned in order to use their oocytes after surviving their disease and with the agreement of their oncologists after an average interval period of 3.4 years. Their mean age at oocyte storing was 35.2 ± 4.1 years (median 35, range 25–41 years). A total of 14 ICSI cycles were carried out. Seventy-three mature oocyte (MII) were warmed with a mean of 6.5 ± 3.5 oocytes for patients (range 4–13). Sixty-three oocytes survived the warming procedure (mean number of 5.7 ± 2.9 for patient, range of 2–10 oocytes). The survival rate of the vitrified-warmed MII eggs was 86.3%. None of the oocytes frozen by slow freezing technique were among those that were thawed. The fertilization rate and normal cleavage rate was 74.6% and 47 embryos were obtained (Table 2). A total of 24 embryos were transferred in 13 cycles all at cleavage stage. The mean number of embryos per patient transferred was 2.2 ± 1.2. Four patients had cryopreserved embryos at blastocyst stage after their warming cycle. The remaining 19 embryos did not reach the blastocyst stage. We obtained four pregnancies, all in breast cancer patients: two miscarriages (one from a cleavage stage transfer and one from a thawed blastocyst transfer) and two live births. The couple pregnancy rate was 36.4% and the delivery rate was 18.2%. Both patients with ongoing pregnancies had a vaginal delivery and live birth at term. The first one had a spontaneous labor at 41 weeks, after a cleavage stage embryo transfer, delivering a female, weighting 3,720 g and a physiological perinatal and maternal outcome. The other patient obtained pregnancy from a thawed blastocyst transfer and had an induced labor at 38 weeks, due to a small reduction of fetal growth, delivering vaginally a female weighting 2,660 g. The perinatal outcome was physiologic, but a maternal light complication due to a post partum uterine bleeding occurred and was treated conservatively. None of the patients had recurrent disease at oncological follow up. Six women out of 244 who performed a procedure of oocyte retrieval have died of their disease. Oocyte storage was not renovated by three patients on the annual phone call service and non-had until now a spontaneous pregnancy. Overall, 95.7% of oocytes are still in deposit as well as two cryopreserved blastocysts.

**Table 1:** Vitrification cycles' characteristics.

**Cancer type**	**Number patients**	**Median age (years)**	**Median number oocytes retrieved**
Breast cancer	146	34.4	11.3
Lymphomas	66	25.9	13.8
Sarcomas	7	27.4	16.1
Ovarian cancer	6	26.8	9
Ematologic	4	18.5	11.7
Colon rectum Ca	4	33.2	8
Cervical cancer	3	34.0	10.3
Others	8	34.1	15.7
**Total**	**244**	**31.4**	**11.8**

**Table 2:** Warming cycles' characteristics and outcomes.

	**Cancer type**	**Age at cryopreservation**	**Age at thawing**	**N thawed oocytes**	**N MII thawed oocytes**	**N used oocytes (MII)**	**N fertilized oocytes**	**N embryos transferred**	**N cryopreserved blastocysts**	**Pregnancy outcome**
1^*^	Lymphoma	35	38	11	8	6	5	4	–	Negative
2	Cervical Ca	38	39	8	6	6	5	2	–	Negative
3	Breast Ca	35	37	9	4	4	2	2	–	Negative
4	Breast Ca	35	38	5	5	2	1	0	–	
5	Thyroid Ca	41	42	5	4	3	3	1	–	Negative
6^*^	Breast Ca	37	41	10	8	8	6	3	1	Pregnancy from thawed blastocyst (miscarriage)
7	Sarcoma	32	39	4	4	3	2	2	–	Negative
8	Breast Ca	25	29	9	9	8	8	2	1	Negative
9^*^	Breast Ca	36	40	15	13	10	8	4	1	Delivery from thawed blastocyst
10	Breast Ca	35	40	12	9	10	4	2	1	Delivery
11	Breast Ca	38	41	5	3	3	3	2	–	Pregnancy (miscarriage)

* *Patients who underwent two warming cycles*.

## Discussion

Fertility preservation has become an emerging assisted reproduction branch that provides women with the possibility of motherhood using their own gametes after age-related decline in fertility or after antineoplastic therapies for cancer (25, 26). In Italy, as in other countries, fertility preservation is free of charge for all oncological women facing treatments with risk of subsequent sterility. According to current international guidelines, fertile women diagnosed with cancer should receive timely reproductive counseling, early in the process of scheduling treatments with a potentially negative impact on their reproductive outcome (27). The rate of women who were offered fertility preservation counseling is not available in our data set. Chung and colleagues in their survey among 457 clinicians in various public hospitals in Hong Kong, reported that only 45.6% were familiar with fertility preservation (28). The factors considered most important for referral were prognosis, patient's motherhood desire, time available before starting gonadotoxic treatment, type of cancer, and type of antiblastic therapy. Most of clinicians who did not refer their patients for fertility preservation reported a lack of available time before treatment, considerable risk of recurrence, poor prognosis, and a lack of awareness of fertility preservation services (7). As Shnorhavorian et al. reported (29), it occurs that patients can search for fertility preservation independently without any oncologist referral, depending on their ethnicity, education, provider type and insurance status. In our population 57.1% of women who underwent fertility counseling turned down the possibility of treatments aimed at saving their fertility potential.

This result may indicate the degree of confidence that physicians have in the process and the level of patients' interest in preserving their fertility. Nevertheless, the number of requests of fertility preservation cycles increased over the years, due to an emerging care from our Cancer Center's clinicians toward their patients' quality of life. This attitude may open new horizons to a change in clinical practice that should consider humanization, psychological issues, and women's quality of life as cornerstones of treatment within any specialty. This is in accordance with patients' interest in the possibility of a future pregnancy and the positive feelings they experienced at the time of fertility preservation. The hope of having a family after a cancer diagnosis can contribute to better acceptance of the oncologic treatment and of its adverse effects (30). Due to these positive psychological aspects and due to the few data in literature about the minimal number of oocytes needed to have a childbirth (31), no limit of ovarian reserve was set for assessing the FP. During fertility preservation counseling the potential limitations, given by the oncological status and the likely number of retrieved oocytes, must be clearly presented for a more aware patients' decision making. At our Institution we have made great efforts to develop a multidisciplinary network aimed at educating patients on their reproductive possibilities after cancer following the American program named Adolescent and Young Adult Health Outcomes and Patient Experiences (AYAHOPE) study (32). Over the years, the efficacy and safety of FP have been improved at our center by the introduction of the use of aromatase inhibitors for women with hormone-sensitive breast cancer (33, 34) and implementation of random-start COS protocols (35–38).

In our study only 4.5% of patients returned to attempt a pregnancy. We observed similar results in male cancer patients who underwent FP in our Fertility Center (39). This low return rate can be explained by different reasons. Depending on the nature of cancer, patients can search for pregnancy once they obtain remission or recover from the disease, a process that could take a long time. Some patients do not survive until their healing. For those who survive, important personal evaluations, such as age and partner status, must be taken into account. Data collection on successful pregnancy outcomes is still at the down.

Reports about using vitrified oocytes in cancer patients are scarce. The first live birth was described in 2007 (40) and in 2008 there was the first birth of twins in a cancer patient with her own frozen oocytes retrieved before bilateral ovariectomy (41). Available data about pregnancies from these gametes are mainly taken from single case reports, except from the analysis by Martinez et al. (26), who described seven pregnancies in their data set. Moreover, the predictable live birth rate model given to oncological patients is based only on data from infertile women, not from this specific group.

Our results add information to the previous limited retrospective reports about reproductive and obstetric outcomes after oocyte vitrification for oncological fertility preservation (42) and is at the best of our knowledge the first complete report in our country. Center storing oocytes must continue to perform vitrification and warming cycles for other indications, due to the long learning curve needed to obtain standard success rates and to the reported volume activity need to mansion this performance (43).

In conclusion, a continuous updated state of knowledge from the latest scientific publications is needed to provide a better tailored counseling and protocol for FP. Increasing the number of collected data and filling registries on reproductive and obstetric outcomes among cancer survivors using their previously cryopreserved oocytes is the key to improve our awareness on the present state of art. This effort can help us to identify the limits of the procedures and can lead to answer the still open question if it is acceptable to limit the access to low prognosis patients or if it is still considered important for their psychological support.

## Data Availability

The datasets generated for this study are available on request to the corresponding author.

## Ethics Statement

This study was approved by the Independent Ethical Committee of the Humanitas Institutional Clinic (Milan, Italy). Consent was obtained from each patient after full explanation of the purpose and nature of all procedures used.

## Author Contributions

CS and AB wrote the research project and analyzed data. VI, CR, AC, and AS contributed to data extraction, references, and follow up analysis. GS contributed to the manuscript preparation. PL-S prepared the final draft.

### Conflict of Interest Statement

The authors declare that the research was conducted in the absence of any commercial or financial relationships that could be construed as a potential conflict of interest.
